# Chloral in Dysentery

**Published:** 1878-11

**Authors:** 


### EXCERPTA.

Chloral in Dysentery.—Dr. Wm. L. Newell, in the *Phila. Med. Times*: A weak solution of that valuable medicine on chronic ulcers manifested such favorable results in my hands that I conceived the idea of using it locally on the inflamed and congested bowel in dysentery. The first case had been under the usual treatment for three days without relief. The child, aged eleven, was tormented with thirst, pain and tenesmus, with twenty-five or thirty dejections in twenty-four hours. In connection with other treatment I ordered five grains of chlor. hyd., dissolved in two ounces of starch gruel, thrown up the bowel with considerable force from a hard rubber syringe. It remained three hours, during which the child slept. Many of the other symptoms were modified and the injection was repeated which remained seven hours, when it came away with some fecal matter, but without tenesmus. The child asked for food, which was given in the form of mutton tea, thickened with boiled wheat flour. All treatment ceased in forty-eight hours from the first enema, four being given in all. The case seemed so satisfactory that I mentioned it to my confrere, Dr. J. S. Whitaker, who has pursued the same treatment with the most happy results in every case, aborting the disease within a few hours. I may mention that he used ten grains instead of five with a lady, aged twenty-five years, who had twenty or thirty calls in twenty-four hours, with complete repose for eight consecutive hours, with permanent abatement of all other symptoms, without other treatment.—*The Medical Brief.*

